# Multimodal cerebral imaging study on the effects of “Adjust Zang Dredge Meridian” electroacupuncture on cerebral central sensitization in PDPN patients: a study protocol for a sham-controlled, randomized trial

**DOI:** 10.1186/s13063-021-05773-w

**Published:** 2021-11-13

**Authors:** Mengyuan Li, Lin Yao, Haipeng Huang, Liying Zhang, Haizhu Zheng, Guan Wang, Zhen Zhong, Shiqi Ma, Shuo Yu, Hongfeng Wang

**Affiliations:** Changchun University of Chinese Medicine, No.1035 Boshuo Road, Nanguan District, Changchun, 130117 Jilin China

**Keywords:** Electropuncture, Painful diabetic peripheral neuropathy, Central sensitization of the brain, Functional magnetic resonance imaging; Clinical trial

## Abstract

**Background:**

Painful diabetic peripheral neuropathy (PDPN) has a great impact on an individual’s quality of life. The current researchers’ previous trial suggested that acupuncture was a promising adjunctive treatment for PDPN. However, the underlying mechanism of action of acupuncture treatment for PDPN is still unclear, especially its effects at the cerebral level. The aim of this trial will be to explore how acupuncture works in treating PDPN by using multimodal cerebral imaging.

**Method and design:**

This will be a randomized controlled trial. A total of 150 participants will be recruited and assigned to one of three groups: the healthy group, the DM without PDPN group and the DM with PDPN group. Participants in the DM without PDPN and the DM with PDPN groups will each be further divided between an electroacupuncture group and a sham electroacupuncture group. Participants will receive six treatment sessions per week for 4 weeks. Multimodal cerebral imaging includes resting-state functional magnetic resonance imaging (rs-fMRI), diffusion tensor imaging (DTI) and magnetic resonance spectroscopy (MRS); this neurophysiological testing will be the primary outcome measure. Subjective pain scales and blood analysis will be a secondary outcome measure and will be used to assess the clinical efficacy of the intervention. Multimodal cerebral imaging will be used to detect cerebral activity changes in each treatment group. The clinical data and fMRI data will be analysed for all the groups. Multiple correlation regression analyses will be used to assess the association between changes in cerebral functional activity and the improvement of clinical outcomes after acupuncture treatment.

**Discussion:**

This study is based on the results of the researchers’ previous study, and using combined clinical and cerebral function changes, it will help evaluate the effects of acupuncture on PDPN. The investigation of acupuncture’s central mechanism of action will further expand the understanding of acupuncture treatment of PDPN.

**Trial registration:**

Chinese Clinical Trial Registry ChiCTR1900024109. Registered on 26 June 2019.

**Supplementary Information:**

The online version contains supplementary material available at 10.1186/s13063-021-05773-w.

## Background

Diabetes mellitus (DM) and its complications are serious diseases that harm human health. In 2016, there were approximately 415 million DM patients in the world, or about 8.8% of the global population [1]. In 2019, the 9th IDF Diabetes Atlas, released by the International Diabetes Federation (IDF), shows that there were 116.4 million patients with DM in China, which now ranks as first in the world in the number of DM patients, and this number is expected to reach 142.7 million by 2045 [2]. Diabetic peripheral neuropathy (DPN) is one of the most common complications of DM. In China, approximately 46.6% of DM patients have DPN [3]. Among DPN patients, painful diabetic peripheral neuropathy (PDPN) is a disease that currently lacks effective treatment [3] and has a serious negative impact on a patient’s quality of life [4, 5]. Approximately 11.4% and 40.5% of patients experience severe and moderate pain, respectively, and of these, 3.7% have lower limb ulcers or even required amputations [6]. More than 2/3 of PDPN patients experience varying degrees of problems with depression and anxiety [7, 8], which pose a burden on their families and on society.

PDPN is characterized by distal symmetric polyneuropathy, and the main clinical manifestations are numbness, tingling and abnormal sensations at the end of limbs, which usually start in the lower limbs and are aggravated at night. The pathogenesis of PDPN is very complicated and includes metabolic disorders, vascular injury, nerve injury, oxidative stress and inflammatory responses. In recent years, researchers have generally assumed that peripheral sensitization and central sensitization play an important role in the production and maintenance of PDPN [9–11]. Studies have confirmed [12] that simple peripheral sensitization is not a key factor leading to the occurrence of PDPN. Hyperalgesia and hypersensitivity can still occur after electrical stimulation of the damaged area following anaesthesia. Therefore, central sensitization should be considered a common and core pathological change in PDPN.

In recent years, the rapid development of multimodal cerebral imaging has become an important method in diagnosing nervous system diseases in vivo. Functional magnetic resonance imaging (fMRI) has been widely used in the study of PDPN, and it provides an exact imaging basis for the study of cerebral structure and changes in functional connections [13, 14]. At present, modern medicine is developing new treatment interventions for PDPN, such as blood glucose control, nerve nutrition and pain-management regimens. Although these methods can relieve patients’ pain and other symptoms to varying degrees, it is still difficult to achieve satisfactory treatment effects and to improve patients’ physical symptoms, quality of life and safety [4]. Acupuncture, as an in vitro treatment method with neurological effects, shows unique advantages in the treatment of PDPN. A number of clinical studies have confirmed that acupuncture can significantly improve pain symptoms in patients with PDPN [15, 16]. In recent years, fMRI has also been applied to study the central regulatory mechanism of acupuncture’s effects [17].

“Adjust Zang Dredge Meridian” electroacupuncture is a therapy established by our research group under the guidance of Chinese acupuncture and the theory of meridian Zang Fu; it incorporates our understanding of the aetiology and pathogenesis of diabetes and its complications. In the pathogenesis of PDPN, seven acupoints have been identified, including Feishu (BL13), Pishu (BL20), Shenshu (BL23), Hegu (LI4), Zusanli (ST36), Sanyinjiao (SP6) and Taichong (LR3). In contrast to the classic acupuncture method of manual twirling, electroacupuncture is safer and more effective in providing pain relief. The combination of classic and electroacupuncture can achieve the effects of regulating zang-fu organs, nourishing meridians and regulating qi and tong jing zhi tong. Based on earlier studies, our research group has demonstrated in both clinical and animal experiments that “Adjust Zang Dredge Meridian” electroacupuncture can effectively improve pain thresholds and nerve conduction velocities in DPN [18, 19]. Therefore, in applying our previous research to the pathological changes of PDPN, we intend to use fMRI technology to explore the potential effects of acupuncture’s central mechanism in the treatment of PDPN. In this proposed study, the first objective will be to use fMRI technology to reveal the mechanisms of central sensitization in the brain of PDPN patients. The second objective will be to investigate the correlation between peripheral neuropathy, subjective pain and changes in brain function in patients with PDPN. The third objective will be to analyse the neuromodulatory effect of “Adjust Zang Dredge Meridian” electroacupuncture on the central sensitization of the brain in PDPN patients through a controlled study of electroacupuncture and sham electroacupuncture and thus to reveal “Adjust Zang Dredge Meridian” electroacupuncture’s mechanism of action in improving the central sensitization of the brain in PDPN patients.

## Method

### Study setting/design

**Fig. 1:**
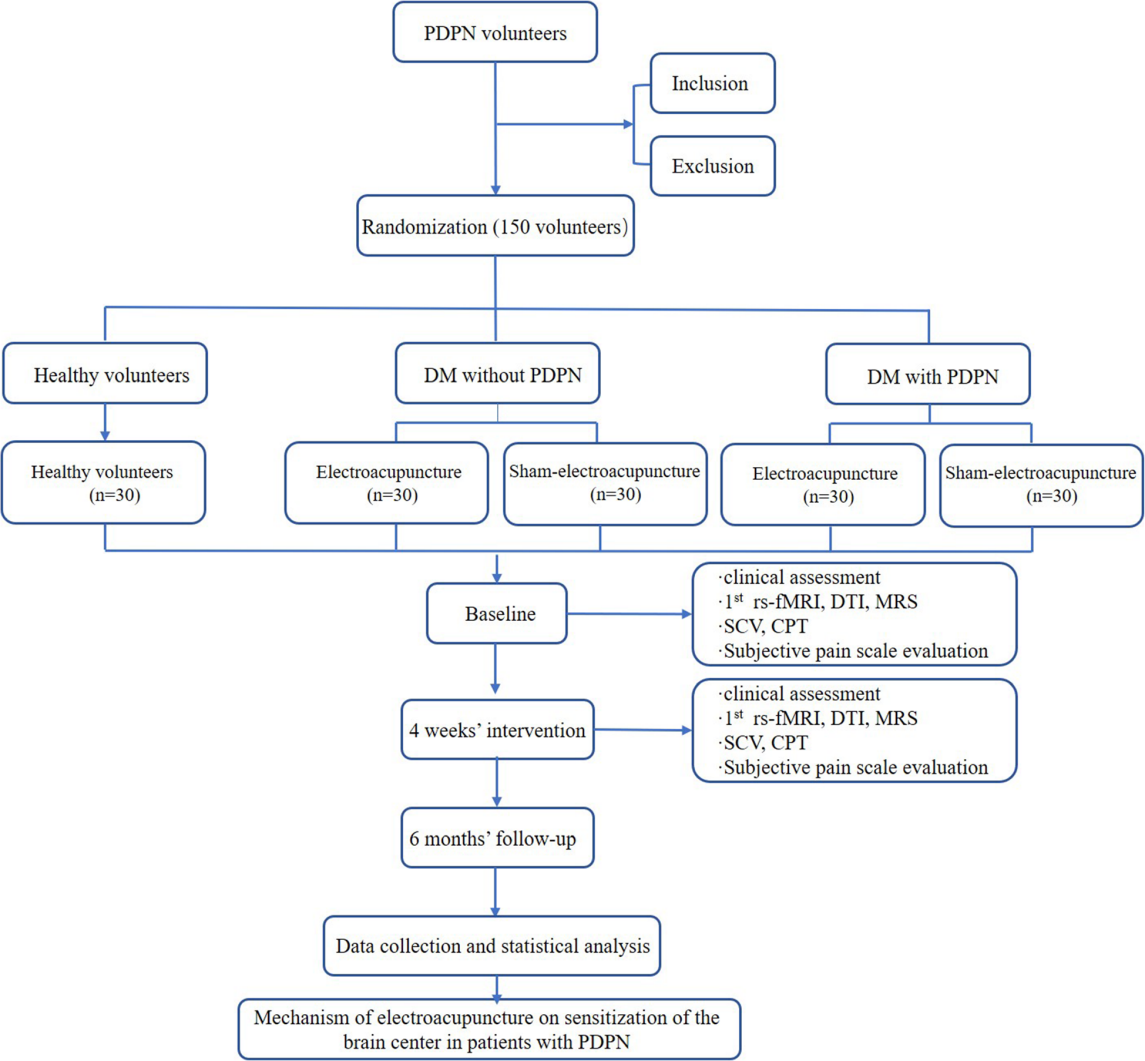
Flow chart of the study trial. PDPN

**Fig. 2:**
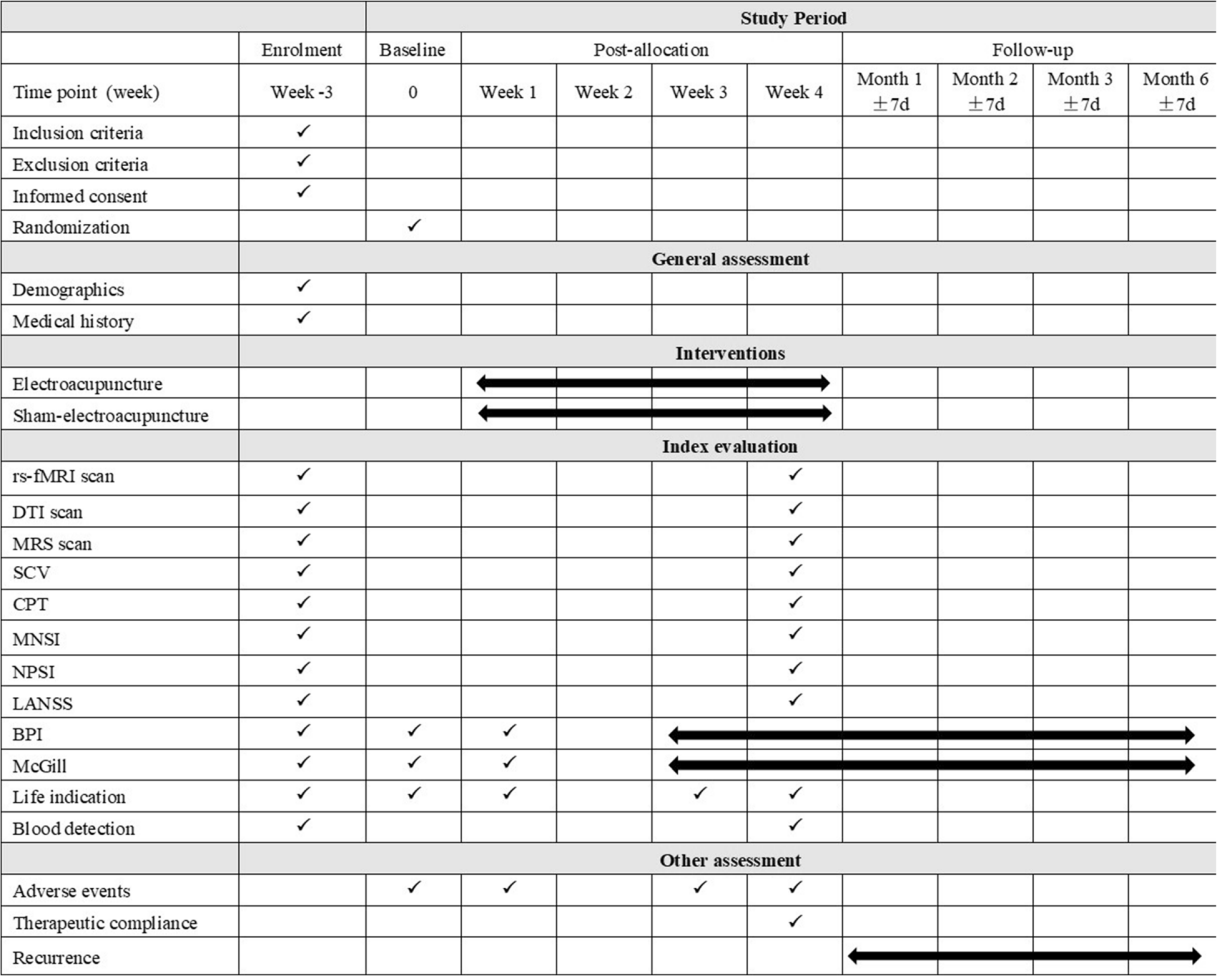
Study schedule

This two-armed randomized, sham-controlled clinical trial will be performed at Guang’anmen Hospital (China Academy of Chinese Medical Sciences) and the Affiliated Hospital of Changchun University of Traditional Chinese Medicine. The study protocol has been approved by ethics committees at both participating hospitals. The clinical trial has been approved by the Ethics Committee of the Affiliated Hospital of Changchun University of Traditional Chinese Medicine (CCZYFYLL2018-002) and by Guang’anmen Hospital (China Academy of Chinese Medical Sciences) (2019-070-KY-02), and it was registered with ChiCTR of the Chinese Clinical Trial Registry (NO: ChiCTR1900024109). A flow chart of the trial is shown in Figs. 1 and 2. This study will be reported based on the STandards for Reporting Interventions in Clinical Trials of Acupuncture (STRICTA) checklist [20] and the Standard Protocol Items: Recommendations for Interventional Trials (SPIRIT) checklist [21] (Table 1 and Additional file 1).

**Table 1:** STRICTA 2015 checklist of acupuncture treatment detail

Item		Detail
1. Acupuncture rationale	1a) Style of acupuncture	Electroacupuncture based on traditional Chinese medicine
	1b) Reasoning for treatment provided, based on historical context, literature sources, and/or consensus methods, with references where appropriate	Based on consensus of the PDPN, meridian and acupoint theory, clinical articles and experience, experimental articles, traditional acupuncture
	1c) Extent to which treatment was varied	Volunteers will receive traditional standardized treatment
2. Details of needling	2a) Number of needle insertions per subject per session (mean and range where relevant)	14
	2b) Names (or location if no standard name) of points used (uni/bilateral)	Bilateral BL13, BL20, BL23, LI4, ST36, SP6, LR3
	2c) Depth of insertion, based on a specified unit of measurement, or on a particular tissue level	1.0 ± 0.5 cm
	2d) Response sought (e.g. de qi or muscle twitch response)	De qi
	2e) Needle stimulation (e.g. manual, electrical)	Electrical density wave with a frequency of 2 Hz/100 Hz and the current intensity is in the range of 0.1~1.0 mA (Hwatuo, Suzhou, China)
	2f) Needle retention time	30 min
	2 g) Needle type (diameter, length, and manufacturer or material)	A sterilised stainless steel needle (length 25–40 mm, diameter 0.25 mm; Hwatuo, Suzhou, China)
3. Treatment regimen	3a) Number of treatment sessions	4 sessions
	3b) Frequency and duration of treatment sessions	1 sessions per day for 6 day
4. Other components of treatment	4a) Details of other interventions administered to the acupuncture group (e.g. moxibustion, cupping, herbs, exercises, lifestyle advice)	Treat with conventional western medicine, drugs to control blood glucose, nourish nerves and analgesia
	4b) Setting and context of treatment, including instructions to practitioners, and information and explanations to patients	The study will be carried out in the Affiliated Hospital of Changchun University of traditional Chinese medicine. All patients will send to the outpatient of acupuncture. Participants will be provided all information except patient allocated group
5. Practitioner background	5) Description of participating acupuncturists (qualification or professional affiliation, years in acupuncture practice, other relevant experience)	A doctor who has a licence with at least 2 years of clinical practice. They can skillfully use acupuncture. All participants received standardized training to ensure consistent treatment
6. Control or comparator interventions	6a) Rationale for the control or comparator in the context of the research question, with sources that justify this choice	Based on consensus of the PDPN, meridian and acupoint theory, clinical articles and experience, experimental articles, traditional acupuncture
	6b) Precise description of the control or comparator. If sham acupuncture or any other type of acupuncture-like control is used, provide details as for items 1–3 above.	For sham electroacupuncture group, besides BL13, BL23 and SP6, LR3 will be stimulated 30 min per day for 6 days using blunt needles that cannot penetrate the skin. Electrical stimulation will perform with the power cord cut off

### Recruitment method

One hundred fifty participants with or without PDPN will be recruited through, but not limited to the review and screening outpatients at Guang’anmen Hospital, social media networking (WeChat), community advertisements (including the distribution of leaflets via regular community health counselling), media campaigns and other networking.

## Recruitment criteria

### Inclusion criteria for participants with DM with PDPN

1. Meet the study’s clinical diagnostic criteria of PDPN;
2. Aged 55–65 years (either sex), with a history of diabetes ≥ 5 years;
3. Present with stable vital signs, with blood pressure, heart rate, blood lipids and other indicators within normal limits;
4. Possess normal cognitive and expressive abilities and have no serious cardiovascular and cerebrovascular disease;
5. Manifest no pre-existing peripheral nervous system lesions;
6. Demonstrate a willingness to provide informed consent, participate voluntarily and cooperate with doctors to complete the clinical trial.

### Inclusion criteria for participants with DM without PDPN

1. Present with a clear history of diabetes ≥ 5 years and a fasting blood glucose (fasting for at least 8 h) ≥ 7.0 mmol/L;
2. Present no symptoms of PDPN;
3. Aged 55–65 years (either sex);
4. Provide a negative CPT test.

### Exclusion criteria

1. Sensitivity to acupuncture and history of experiencing dizziness when exposed to needles;
2. Aged under 55 years or above 65 years;
3. Women who are pregnant or lactating, women preparing for pregnancy, and individuals with a long history of contraceptive use;
4. Signs or symptoms of serious diabetic complications (such as proliferative retinopathy, diabetic nephropathy and the disappearance of dorsal foot artery fluctuation), combined with hyperthyroidism and other diseases that affect blood glucose;
5. The presence of an autoimmune disease;
6. Patients who have participated in other or similar clinical trials within the past 3 months;
7. Individuals with combined cardiovascular and cerebrovascular disease; liver, kidney, haematopoietic and other serious primary diseases; or neuropathy caused by other factors (such as heredity, trauma, alcoholism, drug poisoning, and hypothyroidism);
8. Individuals with a history of recent corticosteroid use for the treatment of other autoimmune diseases;
9. Individuals with a risk for bleeding or who experienced a bleeding event in the last month.

### Healthy participants

1. Aged 55–65 years (either sex), medium size;
2. No restrictive religious beliefs, and no bad habits such as tobacco, alcohol, coffee and tea;
3. Absence of work pressure or life pressure during the observation period;
4. Good health, without any cardiovascular or cerebrovascular diseases or other organic diseases;
5. A negative CPT test.

### Participant withdrawal criteria

1. Subject withdraws voluntarily;
2. Subject experiences serious adverse reactions or serious complications, or a deterioration of the disease occurs in the course of the study, and it is not appropriate to allow continued participation.
3. Subject displays poor compliance with the clinical trial protocols, an unwillingness to continue in the study, or requests to withdraw from the study;
4. Subject is noncompliant with the prescribed treatment, or subject’s incomplete observation data affect the evaluation.

### Treatment of withdrawn participants

1. The researcher will contact each subject by using home visits, telephone calls, letters, etc., and will record the last treatment time and complete the final assessment; the researcher will try to understand the subject’s reasons for withdrawal from the trial. The researcher will truthfully record these data in the case report.
2. The observation data on all withdrawn participants will be obtained by the subject unit at the end of the trial for collection and statistical analysis.
3. Researchers will record the withdrawal information in detail, will report if there is more than one course of treatment, and will follow-up after 3 months.

### Participant discontinuation criteria

If there are serious adverse events, serious complications, or if subjects exhibit deterioration during the trial, the clinical trial will be suspended in accord with a doctor's judgement.

### Treatment of participant discontinuation

1. After a participant is discontinued, the doctor will provide the appropriate clinical treatment.
2. After the termination of a participant, doctors will investigate the cause and maintain detailed records, retain the observation data of the participant and take the final test results as the final result to analyse both the curative effect and adverse reaction data.

### Participant elimination criteria

1. Participants that violate the inclusion and/or exclusion criteria.
2. Participants in erroneous treatment groups.
3. In the exit test, participants documenting the use of banned drugs or treatments and participants with incomplete data collection.

## Random allocation

Eligible participants will be randomly assigned to three groups: healthy participants (*n* = 30), DM without PDPN participants (*n* = 60) and DM + PDPN participants (*n* = 60). The DM without PDPN and DM + PDPN groups will be randomly divided further into an electroacupuncture group (n = 30) and a sham electroacupuncture group (*n* = 30). We will use a computer program (Random Allocation Software, version 1.0; Msaghaei) [22] to generate this random distribution. The total observation period will be 4 weeks. All three groups will receive 24 sessions of either electroacupuncture or sham electroacupuncture treatment over the 4 weeks (six sessions per week during the treatment period). Before the clinical trial begins, the researchers will provide each participant or his/her legal guardian the details of the clinical study. All participants will be required to provide written informed consent before treatment begins and randomization occurs.

## Blinding

During this study, participants and statisticians will be blinded to the group assignments, but the acupuncturists will not be blinded. Before treatment begins, the acupuncturists will be informed of each participant’s identity and group assignment. The participants themselves, however, will be randomly divided into different groups (electroacupuncture and sham electroacupuncture) and will be blind to their assignments.

## Interventions

The acupuncturists providing treatment will be licenced and will have had at least 2 years of clinical experience.

All the acupuncturists who meet this standard will be trained before the trial begins on how to locate acupoints and non-acupoints, how to puncture and how to manipulate needles. Sterile disposable acupuncture needles (length 25–40 mm, diameter 0.25 mm; Hwatuo, Suzhou, China) and SDZ-V electroacupuncture instruments (Hwatuo, Suzhou, China) will be used. Acupuncture will be discontinued if a participant suffers from any adverse events (AEs). During the trial, participants will be prohibited from using treatments other than acupuncture.

## Electroacupuncture

**Fig. 3:**
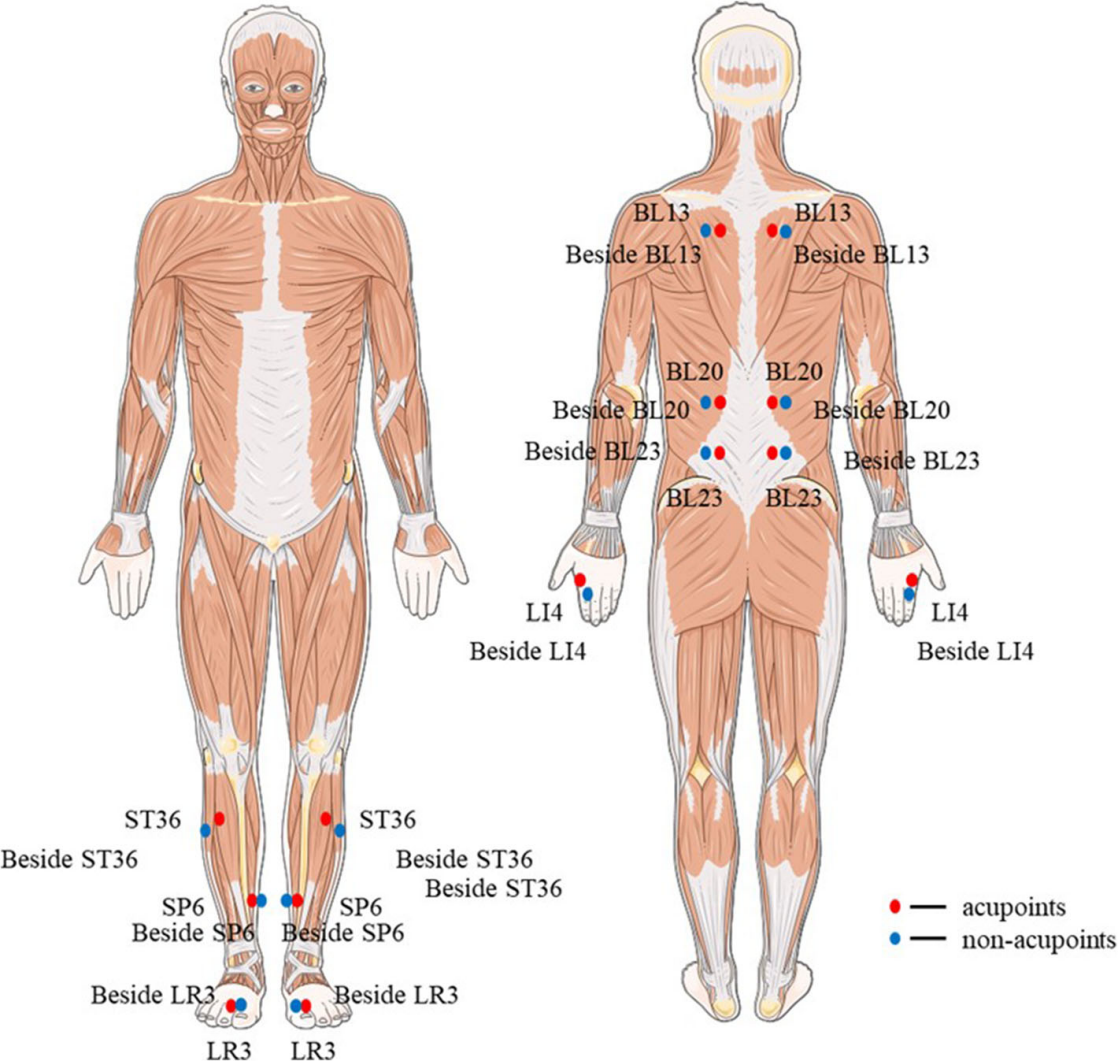
Locations of acupoints and non-acupoints

Participants allocated to the electroacupuncture group will receive treatment with needles inserted at previously designated points. Based on preliminary tests, the research group developed an acupoint matching scheme. The acupoints include bilateral Feishu (BL13), Pishu (BL20), Shenshu (BL23), Hegu (LI4), Zusanli (ST36), Sanyinjiao (SP6) and Taichong (LR3). All acupoints are localized according to the WHO Standard Acupuncture Locations and are shown in Table 2 and Fig. 3. All needles must achieve De qi (a sensation including soreness, numbness, distention, and heaviness), which is believed to be an essential component for acupuncture efficacy. After De qi, unilateral Feishu-Shenshu and Sanyinjiao-Taichong points will be used as the connection points of electroacupuncture. The density wave will be at a frequency of 2 Hz/100 Hz, and the current intensity will be in the range of 0.1~1.0 mA (according to the participant's comfort). A single session will last 30 min/day; 6 sessions will constitute a course of treatment, with 1 day of rest between courses; and the intervention will continue for 4 courses.

**Table 2:** Locations of acupoints in electroacupuncture group

Acupoints	Locations^a^
Feishu (BL13)	On the back, under the spinous process of the 3rd thoracic vertebra, 1.5 cun laterally
Pishu (BL20)	On the back, under the spinous process of the 11th thoracic vertebra, 1.5 cun laterally
Shenshu (BL23)	On the back, under the spinous process of the 2nd lumbar vertebra, 1.5 cun beside the posterior midline
Hegu (LI4)	Between the 1st and 2nd metacarpal bones
Zusanli (ST36)	3 cun directly below Dubi, and one finger-breadth lateral to the anterior border of the tibia
Sanyinjiao (SP6)	Posterior to the mesial border of the tibia, and 3 cun above the tip of the medial malleolus
Taichong (LR3)	In the depression anterior to the junction of 1st and 2nd metatarsal bones

^a^1 cun (≈ 20 mm) is defined as the width of the interphalangeal joint of the volunteer’s thumb

## Sham electroacupuncture

Participants in the sham electroacupuncture group will receive treatment using blunt needles at the designated acupoints. Blunt needles have the appearance of real needles, but they do not penetrate the skin. The sham electroacupuncture group will receive treatment without needle manipulation for De qi, which is characterized by soreness, numbness, heaviness and a feeling of pressure. Non-acupoints are different from real acupoints or meridians and are shown in Table 3 and Fig. 3.

**Table 3:** Locations of non-acupoints in sham-electroacupuncture group

Acupoints	Locations
Beside Feishu	1 cun^a^ beside Feishu (≈ 20 mm)
Beside Pishu	1 cun beside Pishu (≈ 20 mm)
Beside Shenshu	1 cun beside Shenshu (≈ 20 mm)
Beside Hegu	1 cun before Hegu (≈ 20 mm)
Beside Zusanli	1 cun backward Zusanli (≈ 20 mm)
Beside Sanyinjiao	1 cun backward Sanyinjiao (≈ 20 mm)
Beside Taichong	1 cun backward Taichong (≈ 20 mm)

^a^1 cun (≈ 20 mm) is defined as the width of the interphalangeal joint of volunteer’s thumb

The sham electroacupuncture procedure is as follows: the wire to the electroacupuncture instrument is cut off before the intervention. After the acupuncture procedure is completed, the non-acupoints are connected with electroacupuncture, and the parameters and course of treatment will be the same as those in the electroacupuncture group.

Considering ethical factors involved in this sham treatment, we will offer free acupuncture treatment and compensation to participants in the sham electroacupuncture group after all the conclusion of the trial.

## Outcomes

### Primary outcomes

The primary goal of this study is to observe the degree of peripheral neuropathy and measure the subjective pain perceptions in participants with PDPN before and after acupuncture treatment and assess their correlation with the characteristics of the central sensitization state of the brain. We will measure the sensory nerve conduction velocity (SCV) and the current perception threshold (CPT) and will use the McGill Pain Scale while also combining multimodal brain imaging data for our correlation analysis.

#### Cerebral imaging index

The comprehensive application of multimodal cerebral imaging technology includes the use of rs-fMRI, MRS and DTI based on the BOLD effect. Based on the changes in the functional areas of the cerebral cortex, characteristics of functional connectivity in the whole cerebral cortex, functional connectivity between the cerebral cortex and subcortical nuclei and changes to the white matter and metabolic substances in the cerebral cortex, the objective is to analyse the functional and structural changes of cerebral central sensitization and its neuroimaging mechanism in participants with PDPN.

Based on the above results, participants with PDPN and non-PDPN will undergo multimodal cerebral imaging. The data for both groups will be compared and analysed for changes in cerebral functional areas, characteristics of whole cerebral functional connectivity, abnormal white matter and metabolic changes in the cerebral cortex. The aim of the analysis is to understand the mechanism of electroacupuncture in improving cerebral central sensitization in participants with PDPN.

#### Neurophysiological index

The SCV of the bilateral superficial peroneal nerve will be measured by EMG. CPT will be performed at 2000 Hz, 250 Hz and 5 Hz, and for each group, the CPT values on the dorsal side of the first bilateral toe will be measured. The Short-Form McGill Pain Scale will be used to evaluate the subjective pain experienced by the subjects in each group. Multiple regression analysis will be performed to analyse the correlation between the SCV, CPT, McGill Pain Scale values and the multimodal cerebral imaging results to reveal and verify the relationship between the degree of peripheral neuropathy, subjective pain and the changes in cerebral function in participants with PDPN.

Based on the above results, the SCV of the bilateral superficial peroneal nerve, the CPT value of the dorsal side of the first toe and the Short-Form McGill Pain Scale will be recorded and evaluated in participants with PDPN after each electroacupuncture and sham electroacupuncture treatment. We will continue to use the method of multiple regression analysis to analyse the correlation between the SCV, CPT, McGill Pain Scale values and the results of the multimodal cerebral imaging to study the relationship between the clinical therapeutic effect of electroacupuncture and the improvement of cerebral central sensitization and reveal the mechanism of electroacupuncture in the treatment of PDPN.

### Secondary outcomes

Fasting blood glucose, serum C-peptide, blood lipid levels, glycogenesis serum protein, depression and anxiety will be measured in all PDPN participants. All the above measurements will be evaluated at baseline and in the 4th week.

#### Michigan Neuropathy Screening Instrument (MNSI)

The MNSI is simple, convenient and easy to perform, especially for doctors [23]. The MNSI is an effective screening test for diabetic neuropathy because of its accuracy [24]. The MNSI includes two parts: Part A evaluates a participant’s self-assessment of clinical symptoms using 15 “yes” or “no” questions, and part B measures participant symptoms according to a clinical evaluation. The MNSI will be used to assess participants at baseline and in the 4th week.

#### Neuropathic Pain Symptom Inventory (NPSI)

The NPSI is an instrument for evaluating the different symptoms of neuropathic pain [25]. The NPSI is suitable for use in clinical trials because of its good construct validity, its high test-retest reliability and its sensitivity to change. According to the overall impression assessment of participants and examiners on pain changes, the increase or decrease in the total NPSI score is related to improvement or changes in subjective pain. NPSI will be used to assess participants at baseline and in the 4th week.

#### Leeds Assessment of Neuropathic Symptoms and Signs (LANSS)

The LANSS can distinguish patients with neuropathic pain from those with nociceptive pain [26]. It is based mainly on the analysis of sensory descriptions and the inspection of sensory abnormalities. There are 7 questions on the LANSS, and the sensory descriptions include abnormal pain sensation, autonomic nerve dysfunction, tactile pain, paroxysmal pain and skin temperature changes. The evaluation of paraesthesia will include hyperalgesia and acupuncture threshold changes. This method of setting items may increase the discriminative ability of each item. The LANSS will be administered at baseline and in the 4th week.

#### Brief Pain Inventory (BPI)

The BPI is a self-administered questionnaire used to assess pain severity and the effect of pain on the daily function of patients. BPI produces pain severity and pain interference scores ranging from 0 to 10; higher scores indicate worse pain [27, 28]. The BPI has a short and a long form. There are 15 questions on the Short Form BPI (9 questions, with the last question containing 7 parts). In PROMIS calibration testing, 11 of the 15 questions were administered (BPI Items 1, 2, 7 and 8 were omitted). The BPI will be assessed at baseline, the 1st, 2nd, 3rd and 4th weeks of the treatment period, and at the 1-, 2-, 3- and 6-month follow-ups after treatment.

#### Short-Form McGill Pain Questionnaire

The Short-Form McGill Pain Questionnaire is a high-quality tool used to evaluate neuropathic and nonneuropathic pain conditions [29]. It provides basic information for pain research. The questionnaire will be administered at baseline, the 1st, 2nd, 3rd and 4th weeks of the treatment period, and at the 1-, 2-, 3- and 6-month follow-ups after treatment.

### rs-fMRI data acquisition

The rs-fMRI scan will be collected with a 3.0 Tesla superconductor (Skyra, Siemens, Erlangen, Germany) in Guang’anmen Hospital. Participants will undergo rs-fMRI scans at baseline and after the 4th week of treatment. Before the scan, participants will be asked to wear earplugs and remove all metal objects from their bodies. Relevant regulations and procedures of the MRI room will be strictly observed. Using foam pads to reduce head movement, participants will be required to remain supine and awake during the entire scan.

Whole cerebral function imaging adopts a T2-weighted echo planar imaging (EPI) sequence for axial scanning, taking the mid-sagittal parallel connection (AC-PC) as the positioning line. Scanning parameters are as follows: number of layers 47, repetition time (TR) = 3000 ms, echo time = 30 ms, layer thickness = 3.0 mm, field of view (FOV) =220 mm × 220 mm, flip angle = 85°, size of voxel = 3.0 mm × 3.0 mm, in-plane resolution = 64 × 64, phase resolution = 100%, and data collection time = 12:30s.

### DTI scanning

Axial scanning will be performed using a single excitation echo plane imaging (SE-EPI) sequence, taking the mid-sagittal parallel connection (AC-PC) as the positioning line. Scanning parameters are as follows: number of layers 60; repetition time (TR) = 11000 ms; echo time = 98 ms; layer thickness = 2.0 mm; field of view (FOV) = 256 mm × 256 mm; flip angle = 90°; size of voxel = 2.0 mm × 2.0 mm; take 30 directions for the diffusion sensitivity gradient, repeat the measurement twice; diffusion sensitivity coefficient *b* value 1000s/mm^2^; axial scanning obtains no gradient diffusion weighted imaging (*b* = 0); and data collection time = 12:52 s.

### MRS scanning

Hydrogen proton magnetic resonance spectroscopy and 3D multivoxel spectroscopy scanning will be used. Scanning parameters are as follows: repetition time (TR) = 1750 ms, echo time = 144 ms, layer thickness = 5.0 mm, repeat time = 128, and data collection time = 4:24 s.

### Treatment of adverse events (AEs)

While focusing on efficacy, researchers must be alert to the occurrence of adverse reactions or AEs. When an AE is discovered, researchers will take necessary measures to treat the condition until the adverse event resolves. Adverse reactions or AEs, whether related to the study or not, will be recorded in detail, including date of onset, signs and symptoms, examination index, outcome, date of resolution, any actions taken, duration and follow-up. AEs should be resolved within 24 h. The common treatment-related AEs include fainting due to needle stick, subcutaneous haemorrhage, redness and swelling of the acupuncture site and continuous pain at acupuncture site.

### Quality control and assurance

Before this study begins, all researchers will conduct a unified, standardized operation training to ensure that they are proficient in the treatment protocols and evaluation techniques; this training will improve the reliability of the study results. In this study, first, the laboratory examination index and normal value range will be defined, and standard operating procedures (SOPs) will be formulated to strictly control the quality of the laboratory examination index. In the process of rs-fMRI scanning, the magnetic resonance inspection requirements will be strictly followed and will include quality control of the subjects’ scanning state, the scanning equipment, the magnetic field and the image acquisition to ensure the quality and reliability of the study data. During the treatment period, scientific standards of informed consent, active health education, strengthened humane care, long-term follow-up plans and other means to improve subject compliance will be implemented.

According to the relevant requirements of the State Administration of Traditional Chinese Medicine and the study group, clinical research SOPs will be formulated to ensure that established procedures are followed throughout clinical research. The Data Monitoring Committee is composed of members of the Ethics Committee, and the committee has determined that there is no conflict of interest with this experiment. A data acquisition and management system has been set up and includes double entry for two persons, data entry personnel and researchers, to complete the first and second entry, respectively, with a third person checking to ensure the objectivity, authenticity and accuracy of the study data. Quality control of the data will be handled at least once every 4 months.

After blind auditing of the data, we believe that the established database will be correct, and the data will be locked by the main researchers and statisticians. The locked data file will not be allowed to change again. The database will be submitted to statisticians for statistical analysis according to the requirements of the statistical plan.

### Sample size calculation

This proposed study is an RCT whose primary aim is to evaluate the cerebral imaging mechanism of acupuncture to improve cerebral central sensitization and the clinical effect of acupuncture in patients with PDPN. Acupuncture is a complex intervention that differs from other treatments. Its therapeutic effect is changed if the acupoints are altered. No two-arm, randomized, sham-controlled trial has been conducted on this topic. For this reason, we are using our previous clinical experience to calculate the efficacy of electroacupuncture as an intervention [30, 31]. The proposed sample size of this trial was calculated mainly based on the Short-Form McGill Pain Questionnaire and the assumption that after treatment, the average change reported using this questionnaire would be greater for the experimental group than for the control group. The mean difference was 0.81, with a standard deviation of 0.07. Sample size was calculated using a noninferiority comparison. We set the alpha risk (*α*, type 1 error risk) at 0.025, the beta risk (*β*, type 2 error risk) at 0.2 and the critical value (*Δ*) at − 0.1. Calculating by using SAS [32], the sample size result was 24. Thus, according to this sample size calculation, each group should recruit 24 participants. Allowing for a 20% attrition rate in this study, we plan to recruit 150 participants, with 30 in each of the study groups. Healthy participants will not receive any treatment. Each participant will receive 2 rs-fMRI scans to observe different cerebral activities before and after acupuncture treatment.

## Statistical analysis

### Neuroimaging data analysis

Cerebral imaging testing will be entrusted to a third party, professionals of the independent neuroimaging research laboratory at Martinos Center for Biomedical Imaging, Massachusetts General Hospital, Harvard Medical School, USA, for statistical analysis. (1) fMRI data preprocessing: We will use SPM12 for slice timing correction (SPM12, http://www.fil.ion.ucl.ac.uk/spm/), rigid body correction for head motion with the FSL (https://fsl.fmrib.ox.ac.uk/fsl/fslwiki/) [33, 34], subjects with head movement < 1 mm and a rotational shift of < 2° will be entered into the subsequent analysis, bandpass temporal filtering (0.01–0.08 Hz), whole brain signal regression and ventricular and white matter signal regression. We will use the FreeSurfer version 6.0.0 software package to process the structural data and surface mesh representations of the cortex from each participant’s images [35]. Images will be reconstructed and registered to a common spherical coordinate system; functional images will be coregistered to anatomical images and normalized to the MNI152 standard template. (2) Morphological analysis of the grey matter structure of the brain: surface-based morphometry (SBM), voxel-based morphometry (VBM) and morphological analysis of subcortical grey matter structures in the grey matter of the brain will be based on anatomical data. The CIVET-pipeline toolkit and SurfStat software in the MATLAB environment will be used for the processing and statistical analysis of the image data. (3) Analysis of white matter parameters of the brain: Based on DTI scanning, the voxel-based analysis method will be used to process and analyse the test data using the MATLAB toolkit PANDA software. Image processing will include converting images from DICOM format to NIfTI format, eliminating extracerebral tissue and clip aligned images, eddy current correction and realignment, image normalization, calculating tensor and colour maps and fibre tracking and visualization. Finally, FSL software will be used to present the data, and the corresponding white matter fibre bundles will be identified according to the core white matter map of Johns Hopkins University. (4) Brain metabolite analysis: Based on the MRS scanning, images will be converted from DICOM format to RDA format, RDA data will be imported into the LC Model software, GAMMA software will be used in the same sequence and with the same parameters, the absolute concentration of each substance in the imported data will be calculated, and the substance concentration with SD < 15% will be imported into the SPSS 26.0 software package and analysed according to statistical principles. (5) Correlation analysis of SCV, CPT, McGill and multimodal brain imaging research data: Pearson linear correlation analysis in the SPSS 26.0 package will be used to correlate clinical effect indicators such as SCV, CPT and McGill values with the change values from multimodal brain images. The statistical threshold will be set as *P* < 0.05, a Pearson correlation coefficient of *r* > 0 will indicate a positive correlation, and *r* < 0 will indicate a negative correlation. According to the results of the correlation analysis, multiple regression analysis or principal component analysis will be carried out to deeply explore the brain function change characteristics and pathogenesis of brain central sensitization in PDPN.

### Clinical data analysis

According to the study data, the effect indices of the two groups will be calculated and compared to analyse the influence on the prognosis of PDPN. Indices of scales will be analysed by repeating measurement analysis of variance. The main analytical indicators will be the curative effect index and recurrence rate. A *t* test will be used for measurement data with a normal distribution. The Wilcoxon rank sum test will be used for nonnormal distribution; the Pearson chi-square test will be used for counting data. If it is a sequential concatenation table, the CMHX^2^ test will be used.

The measurement data of different treatment groups will be statistically described by the mean ± standard deviation (‾*χ* ± s). Intragroup differences will be assessed using a t-test at baseline; analysis of variance (ANOVA) will be used to compare measurement data at different stages; and SNK will be used for pairwise comparisons. Changes between groups before and after treatment will be analysed by ANOVA. If the data do not fit the above statistical analysis conditions (such as nonnormal), a nonparametric test will be used.

Counting data from different treatment groups are statistically described by frequency (constituent ratio). Changes between groups before and after treatment will be tested using the X2 test (CMHX2 test) or nonparametric test. The analysis of data results from participants not complying with the study protocol will be treated according to statistical analysis with randomization and multiple interpolation.

## Discussion

Among diabetic patients, approximately 25% develop PDPN. The probability of PDPN is higher in patients with type 2 diabetes (T2DM) [36]. In PDPN, which is most often associated with sensory motor neuropathy, patients may feel burning, pricking or electric pain. Some patients may even describe hyperalgesia or abnormal pain [37, 38], which greatly reduces their quality of life. Acupuncture is a nonpharmacological treatment. It is very effective for pain relief, especially for PDPN. A Japanese study showed that 20 patients with PDPN who received acupuncture treatment had better pain relief than the patients in the drug treatment group [39]. Another study confirmed that acupuncture can effectively improve the main symptoms of patients with PDPN, significantly reducing or even ending drug use [40]. There is still a lack of clear reporting on the mechanism of acupuncture in the treatment of PDPN. Acupuncture can act on all parts of the central nervous system, and it can exert analgesic effects by regulating neurotransmitters and signalling pathways and immune responses [41]. Using multimodal cerebral imaging technology, Zhou [42] found that electroacupuncture at Weizhong (BL40) and Dachangshu (BL25) can change the cerebral function pain centre and cerebral default network. Therefore, this clinical trial aims to determine whether acupuncture can improve the state of cerebral central sensitization in patients with PDPN. We will use rs-fMRI to better explore the neuroregulatory effect of acupuncture on PDPN cerebral central sensitization and to improve the overall efficacy of acupuncture.

To date, high-quality clinical evidence for acupuncture in PDPN is still insufficient because most clinical trials have had small sample sizes, short or nonexistent follow-up or other unavoidable restrictions. The design of this study takes into account the placebo effects, so two sham acupuncture groups will be set up for comparison. To avoid deviations in the results and improve the reliability of the clinical data, we will try to maintain the consistency of the baseline. Participants will be strictly selected according to the inclusion and exclusion criteria. The rs-fMRI of all participants will be carried out in the MRI room of Guang’anmen Hospital (China Academy of Chinese Medical Science). Our research will help explore the potential clinical applications of acupuncture.

## Trial status

This trial was registered with the Chinese Clinical Trial Registry (www.chictr.org.cn/) on 26 June 2019. This trial is currently in the recruitment phase. We predict that recruitment work will be finished by December 2021.

## Supplementary Information

**Additional file 1.** SPIRIT 2013 Checklist: Recommended items to address in a clinical trial protocol and related documents.

## Data Availability

Not applicable.

## Abbreviations

DM: Diabetes mellitus
DPN: Diabetic peripheral neuropathy
PDPN: Painful diabetic peripheral neuropathy
rs-fMRI: Resting-state functional magnetic resonance imaging
DTI: Diffusion tensor imaging
MRS: Magnetic resonance spectroscopy
MNSI: Michigan Neuropathy Screening Instrument
NPSI: Neuropathic Pain Symptom Inventory
LANSS: Leeds Assessment of Neuropathic Symptoms and Signs
BPI: Brief Pain Inventory
